# Keeping An Eye Out for Stroke—Herpes Zoster Ophthalmicus Leading to Acute Ischemic Stroke

**DOI:** 10.5811/cpcem.50693

**Published:** 2026-06-29

**Authors:** Summer Burke, Mary Armbruster, Jacqueline Le

**Affiliations:** Desert Regional Medical Center, Department of Emergency Medicine, Palm Springs, California

**Keywords:** herpes zoster ophthalmicus, stroke, myocardial infarction, pulmonary emboli, case report

## Abstract

**Introduction:**

Herpes zoster ophthalmicus, a reactivation of varicella-zoster virus involving the ophthalmic branch of the trigeminal nerve, is a known but under-recognized risk factor for acute cerebrovascular and cardiovascular events.

**Case Report:**

We report the case of an 84-year-old female with herpes zoster ophthalmicus who suffered a fatal ischemic stroke and bilateral submassive pulmonary emboli within days of diagnosis. Despite early identification and appropriate antiviral treatment, the patient experienced rapid neurological decline and ultimately succumbed to complications.

**Conclusion:**

This case underscores the critical association between herpes zoster ophthalmicus and increased risk of stroke and myocardial infarction, as supported by growing epidemiologic evidence. Physicians should be aware of these severe complications, consider close monitoring and cardiovascular risk stratification in patients with herpes zoster ophthalmicus, and they should provide patients with clear return precautions for symptoms suggestive of stroke or embolic disease. Further research is needed to determine preventative strategies beyond antiviral therapy for high-risk patients.

## INTRODUCTION

Over 95% of adults worldwide have been infected with the varicella-zoster virus, and approximately one in three individuals will develop herpes zoster in their lifetime.^1^ Caused by reactivation of the varicella-zoster virus, herpes zoster results in a painful vesicular rash that appears unilaterally on the body in a single or occasionally two contiguous dermatomes. Herpes zoster ophthalmicus (HZO) occurs when the virus specifically affects the ophthalmic branch of the trigeminal nerve, whose dermatome covers some of the scalp, forehead, eyelid, eye, and nose. Herpes zoster ophthalmicus is a vision-threatening condition, as the virus affects the globe of the eye, resulting in keratitis with characteristic dendritic lesions seen with fluorescein staining. The incidence rates of HZO are reportedly between 10–20% of those with herpes zoster.^2^ Research has indicated that HZO leads to a viral vasculitis, with direct infiltration of vascular tissue by the virus and subsequent autoantibody formation that predisposes the patient to the development of stroke and myocardial infarction.^3^,^4^

## CASE REPORT

An 84-year-old female with a history of hypertension and hyperlipidemia presented to the emergency department (ED) of Facility A for right eye and right upper face pain with an associated rash for two days. She noted resolution of an upper respiratory infection over the prior two weeks. Vital signs were unremarkable, and physical examination was notable for a vesicular rash distributed in a V1 dermatomal pattern on the right face. Pupils were equally reactive, and visual acuity was 20/40 in each eye. Slit-lamp and fluorescein examination revealed a dendritic lesion at the three o’clock position on the right cornea. The patient was diagnosed with HZO, and as recommended by the consulting ophthalmologist over the phone, she was given an intravenous dose of acyclovir, a seven-day prescription of oral acyclovir, and office follow-up the next morning.

The following day, the patient was unable to see ophthalmology as planned and presented to the ED of Facility B with the same complaints. Vital signs were unremarkable, and physical examination was consistent with the HZO findings seen the previous day. Neurological examination was grossly intact. Another ophthalmologist consulted at that time recommended outpatient follow-up within a few days and to continue the prescribed acyclovir regimen. A case manager helped to ensure this follow-up.

**Image 1:**
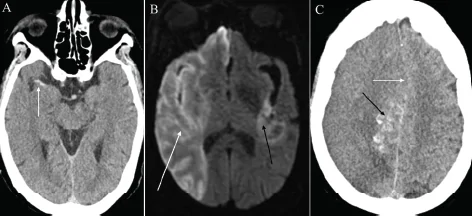
Axial views of the patient’s brain imaging over a period of 24 hours. (A) Initial computed tomography (CT) of the head without contrast revealed a hyperdense right middle cerebral artery (arrow). (B) Diffusion-weighted magnetic resonance imaging of the brain showed acute infarct in nearly the entire right middle and anterior cerebral artery territories (white arrow), as well as acute infarct of the left insular cortex and temporal lobe of the left middle cerebral artery territory (black arrow). (C) Subsequent CT head without contrast showed hemorrhagic transformation with intraparenchymal hemorrhage (black arrow) and 7 mm of midline shift (white arrow).

**Image 2:**
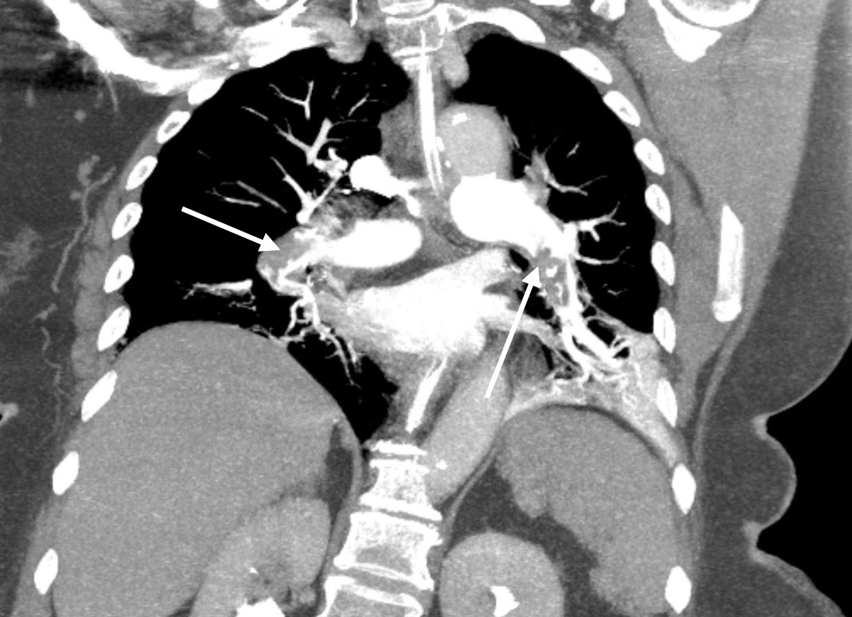
Computed tomography angiography of the chest (coronal view) showing extensive bilateral pulmonary emboli (arrows) in an elderly patient diagnosed with herpes zoster ophthalmicus.

Three days later, the patient was found unresponsive at home with a rightward gaze, left facial droop, left-sided paralysis, and Glasgow Coma Scale (GCS) 4. Neurological examination was limited, and the patient was intubated by emergency medical services enroute to Facility A’s ED. Computed tomography (CT) of the head without contrast revealed a hyperdense right middle cerebral artery (MCA) and loss of gray-white matter differentiation in the right MCA territory (Image 1A). Computed tomography angiography (CTA) of the head and neck revealed occlusion of the right M1 and A2 segments and, incidentally, large bilateral pulmonary emboli (PE). A CTA chest subsequently showed extensive bilateral PE in the main, lobar, and segmental pulmonary arteries with evidence of right heart strain (Image 2).

**Image 3:**
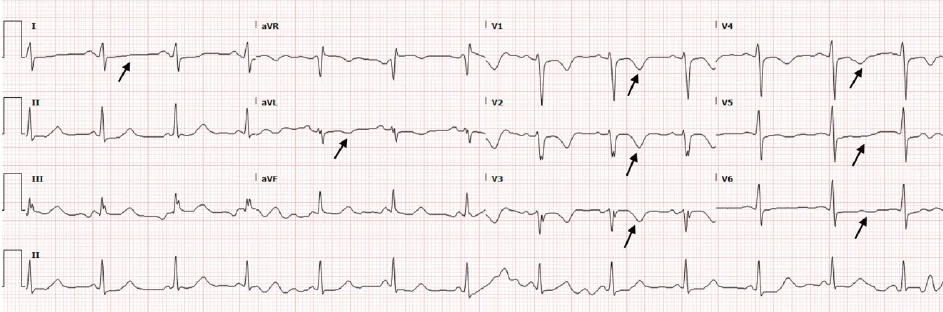
Electrocardiogram showing abnormal T-wave inversions and flattening suggestive of anterolateral ischemia (arrows) in an elderly patient diagnosed with herpes zoster ophthalmicus.

Laboratory blood tests were unremarkable except for an elevated troponin level of 1.05 nanograms per milliliter (ng/mL) (reference range: 0.0–0.03 ng/mL). Electrocardiogram demonstrated moderate T-wave abnormalities consistent with anterolateral ischemia (Image 3). Although she was outside the window for thrombolytic therapy for her MCA stroke, transfer to Facility B was initiated for possible mechanical thrombectomy. The interventional neurologist at Facility B felt treatment for the PE should take precedence, and a heparin drip was initiated prior to transfer.

*CPC-EM Capsule*

What do we already know about this clinical entity?

*Herpes zoster ophthalmicus can lead to serious ocular complications including vision loss.*

What makes this presentation of disease reportable?

*This case highlights the under-recognized association between herpes zoster and acute cerebrovascular and cardiovascular events.*

What is the major learning point?

*Acute herpes zoster ophthalmicus infections can increase the risk for deleterious vascular sequelae, including stroke and myocardial infarction.*

How might this improve emergency medicine practice?

*Recognition of potential complications will promote prompt antiviral treatment, patient education on the early signs of vascular insults, and close follow-up.*

On arrival to the ED of Facility B, the patient’s neurological exam improved to a GCS 6T, and she was seen reaching for her endotracheal tube with her right arm. Pupils were sluggish but reactive and symmetric bilaterally. Repeat CT head showed decreased gray-white differentiation in the right MCA territory, as well as a hyperdensity in the right basal ganglia concerning for new hemorrhage versus contrast staining from prior CTA. Heparin drip was paused due to concern for cerebral hemorrhage along with a supratherapeutic partial thromboplastin time (PTT) of >139 seconds (therapeutic goal 52–72 seconds, reference range: 22–29.5 seconds). The neurology team recommended against thrombectomy based on the above CT findings indicative of a completed stroke. Neurosurgery’s evaluation showed an improved GCS 9T, continued left-sided flaccid paralysis, localization to pain on the right, right-sided gaze preference, and equally reactive pupils bilaterally.

The patient was subsequently admitted to the intensive care unit. Repeat troponin level was unchanged from prior. Echocardiogram revealed right heart strain and no evidence of structural anomalies that might result in paradoxical embolism. Subsequent PTT improved to 57.2 seconds four hours after the initial result, and heparin drip was restarted. The patient was started on levetiracetam for seizure prophylaxis, valacyclovir for HZO, and propofol and fentanyl drips for sedation and comfort.

Diffusion-weighted magnetic resonance imaging of the brain with and without contrast obtained overnight revealed findings consistent with acute ischemic infarction involving nearly the entire right MCA and anterior cerebral artery (ACA) territories, with prominent areas of hemorrhage within the right basal ganglia and medial right frontal parietal cortex and subcortical area. A new hemorrhagic infarct within the left MCA territory centered within the insular and temporal area was also seen (Image 1B). The heparin drip was again discontinued. The next morning, the patient’s neurologic exam declined to GCS 6T, and a repeat CT head revealed increased edema in the right MCA and ACA territories, new petechial hemorrhage in the right putamen and distal right ACA territories, and new edema and petechial hemorrhage in the left frontal lobe, with a hyperdense adjacent left M3 branch visualized. New mass effect with sulcal and ventricular effacement and seven millimeters of midline shift were noted (Image 1C). The patient had not yet exhibited signs of herniation, and 3% normal saline infusion was initiated. She was not a candidate for neurosurgical decompression secondary to her age and overall poor prognosis. The following day, the patient’s family placed the patient on comfort measures. She expired the following evening, three days after admission.

## DISCUSSION

The pathogenesis of HZO leading to stroke is not definitive, although studies on brain tissue from individuals suffering a stroke within 10 months of cranial or cervical herpes zoster onset suggest spread of the virus transaxonally to the cerebral arteries, affecting them from outside-in, starting with inflammation of adventitia and transmurally extending to intima. This inflammation and remodeling predisposes individuals to stroke and ischemia.^3^,^4^ Multiple studies have identified and highlighted the link between herpes zoster and increased stroke risk.^4^–^8^ In a meta-analysis, relative risk of stroke at two weeks following herpes zoster was 2.36.^6^ Stroke risk for HZO remained increased for the first year following diagnosis, and risk has been shown to increase with age and immunocompromised states.^1^,^8^

While the link between HZO and stroke as well as myocardial infarction (MI) has been studied and documented,^4^–^8^ it is not yet well-recognized as a potential complication in the emergency medicine community. This case highlights lesser known adverse outcomes of what has been considered a less dangerous condition in terms of mortality. The exact effectiveness of vaccination and aggressive antiviral treatment on subsequent stroke risk is inconclusive, but they remain promising prevention and treatment strategies.^1^,^4^,^5^,^7^,^8^ To date, there are no published studies on other interventions that may reduce the likelihood of stroke or MI following a herpes zoster outbreak, such as possible initiation of antiplatelet or anti-inflammatory therapy.

This patient also developed pulmonary emboli, which to date have not been reported in conjunction with HZO. There is, however, a case report of pulmonary thromboembolism following herpes zoster oticus.^9^ Furthermore, two studies in children with varicella demonstrated transient elevations in lupus anticoagulant, as well as autoantibodies to protein S, protein C, prothrombin and antithrombin; and those with thromboembolism consistently had elevated autoantibodies to protein S and lupus anticoagulant.^10^,^11^ While these studies were limited to children and acute varicella infections as opposed to reactivated zoster infections, it may be reasonable to consider similar biologic responses to the same virus. Additionally, a bench study found pro-thrombotic, platelet activating and aggregating viral exomes in those with acute herpes zoster infections.^12^ It is possible these findings are contributing factors to herpes zoster-associated vasculopathy, stroke, and MI; and in our case, may have contributed to the development of pulmonary emboli. Further research is required to definitively establish a hematologic-based pro-thrombotic mechanism caused by herpes zoster.^2^

## CONCLUSION

This case highlights the risk of significant vascular sequelae, including acute stroke and MI, following herpes zoster ophthalmicus infection. With the risk of subsequent cerebrovascular and cardiovascular disease increased after HZO infection, additional research is needed to identify preventative strategies and interventions to reduce these potentially devastating complications. We encourage physicians to consider close follow-up and cardiovascular risk stratification in patients with HZO. Physicians should provide risk-mitigation strategies, such as routine vaccination against herpes zoster for those at high risk for vascular events, and clear return precautions for symptoms suggestive of stroke or embolic disease.
